# The Tricarboxylic Acid Cycle Metabolites for Cancer: Friend or Enemy

**DOI:** 10.34133/research.0351

**Published:** 2024-06-12

**Authors:** Jie Wu, Nian Liu, Jing Chen, Qian Tao, Qiuqiu Li, Jie Li, Xiang Chen, Cong Peng

**Affiliations:** ^1^ The Department of Dermatology, Xiangya Hospital, Central South University, Changsha, Hunan, China.; ^2^ Furong Labratory, Changsha, Hunan, China.; ^3^ Hunan Key Laboratory of Skin Cancer and Psoriasis, Hunan Engineering Research Center of Skin Health and Disease, Xiangya Hospital, Central South University, Changsha, Hunan, China.; ^4^ National Engineering Research Center of Personalized Diagnostic and Therapeutic Technology, Changsha, Hunan, China.; ^5^ National Clinical Research Center for Geriatric Disorders, Xiangya Hospital, Central South University, Changsha, Hunan, China.

## Abstract

The tricarboxylic acid (TCA) cycle is capable of providing sufficient energy for the physiological activities under aerobic conditions. Although tumor metabolic reprogramming places aerobic glycolysis in a dominant position, the TCA cycle remains indispensable for tumor cells as a hub for the metabolic linkage and interconversion of glucose, lipids, and certain amino acids. TCA intermediates such as citrate, α-ketoglutarate, succinate, and fumarate are altered in tumors, and they regulate the tumor metabolism, signal transduction, and immune environment to affect tumorigenesis and tumor progression. This article provides a comprehensive review of the modifications occurring in tumor cells in relation to the intermediates of the TCA cycle, which affects tumor pathogenesis and current therapeutic strategy for therapy through targeting TCA cycle in cancer cells.

## Introduction

Glucose is a vital nutrient that serves as a primary source of energy and carbon and relies on glucose transporters (GLUTs) to enter cells. In the cytoplasm, one molecule of glucose undergoes a series of reactions known as glycolysis, leading to its conversion into 2 molecules of pyruvate [1]. When there is insufficient oxygen supply or impaired oxygen utilization, pyruvate is further catalyzed to lactate. With adequate oxygen supply, pyruvate primarily enters the mitochondria and completely oxidates to carbon dioxide and water via the tricarboxylic acid (TCA) cycle.

**Fig. 1.**
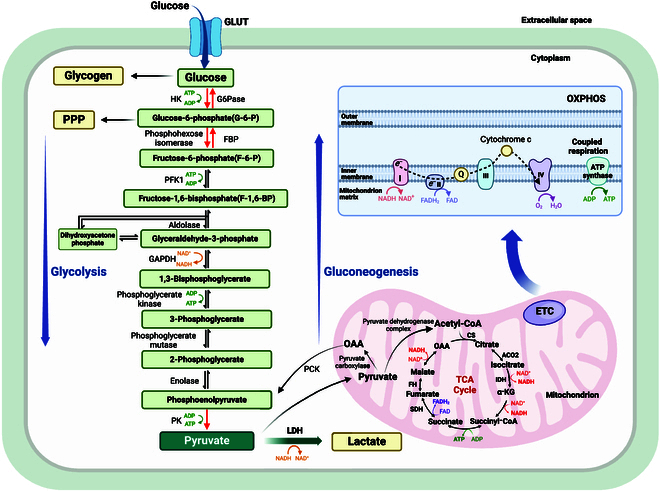
Overview of glucose metabolism and OXPHOS. In the cytoplasm, glucose converts into pyruvate through glycolysis or synthesizes glycogen. Glucose is phosphorylated by HK to produce glucose-6-phosphate (G-6-P). The reaction is irreversible and consumes one molecule of ATP. G-6-P is converted to fructose-6-phosphate (F-6-P) catalyzed by phosphohexose isomerase or enters the pentose phosphate pathway (PPP). F-6-P converted to fructose-1,6-bisphosphate (F-1,6-BP) catalyzed by PFK1, which is also irreversible and consumes ATP. F-1,6-BP is catalyzed by aldolase to produce glyceraldehyde-3-phosphate and dihydroxyacetone phosphate, which are isomers that can be converted and supplemented by each other. Glyceraldehyde-3-phosphate is oxidized to 1,3-diphosphoglycerate by glyceraldehyde-3-phosphate dehydrogenase (GAPDH), and NAD^+^ accepts hydrogen and electrons as a coenzyme. Phosphoglycerate kinase catalyzes substrate-level phosphorylation of 1,3-diphosphoglycerate to produce ATP and 3-phosphoglycerate. 3-Phosphoglycerate converts to phosphoenolpyruvate (PEP) through 2-step reactions. PEP undergoes substrate-level phosphorylation to produce pyruvate and ATP catalyzed by PK, which is irreversible. Pyruvate can produce glucose in the opposite direction of glycolysis. There are only 3 differences in irreversible reactions. Pyruvate enters the mitochondria to transform into OAA and back into the cytoplasm to form PEP. F-6-P is catalyzed by FBP to produce G-6-P, which produces glucose catalyzed by glucose-6-phosphatase (G6Pase). Under hypoxia condition, pyruvate is catalyzed by LDH to produce lactate. Under normal oxygen conditions, pyruvate is transferred to the mitochondria to produce acetyl-CoA and then enters the TCA cycle. NADH and FADH2 gradually lose electrons through the electron respiratory chain and are oxidized to water. Complex I transfers electrons from NADH to ubiquinone, while complex II transfers electrons from succinate to ubiquinone. Complex III transfers electrons from reduced ubiquinone to cytochrome c and subsequently to oxygen through complex IV. The electron transport process is accompanied by the gradual release of energy, which drives the phosphorylation of ADP to ATP. The oxidation of NADH and FADH2 is coupled with the phosphorylation of ADP, which is called OXPHOS.

The TCA cycle, also known as the citric acid cycle or Krebs cycle, serves as a central hub for the oxidative metabolism of glucose and other fuel molecules in mitochondria [2]. The TCA cycle begins with the entry of acetyl-coenzyme A (CoA) produced by glycolysis, the β-oxidation of fatty acids or the amino acid catabolism [3]. The TCA cycle undergoes a series of redox reactions to generate high-energy electrons that carried by reduced form of nicotinamide adenine dinucleotide (oxidized form) (NAD^+^) (NADH) and flavin adenine dinucleotide (FADH2) to enter the electron transport chain (ETC). Utilizing energy from electrons, the formation of an electrochemical gradient mediated by protons transportation drives the synthesis of substantial adenosine 5′-triphosphate (ATP) through oxidative phosphorylation (OXPHOS) [4]. Other than that, the TCA cycle provides other metabolic pathways with vital intermediates (Fig. 1).

**Table.** The effect of TCA cycle intermediates on cancer

**Intermediates**	**Effect on cancer**	**Mechanisms: Signaling pathway/metabolic rewiring**	**References**
**Citrate**	Inhibiting cancer	Promoting gluconeogenesis and a shift from glycolysis to OXPHOS by activating FBP	[32]
		Suppressing glycolysis by inhibiting PFK1/2, F1,6BP, and LDHA	[30,33,35]
		Inducing apoptosis via activating caspase-2/3/8, the cleavage of ADP-ribose polymerase, the release of cytochrome c, and inhibiting Mcl-1	[38,39]
		Activating autophagy through down-regulating the CaMKII/ AKT/mTOR pathway	[40]
		Inducing pyroptosis through caspase-4/NLRP3/GSDMD pathway	[41]
		Excess lipid biosynthesis and cellular senescence	[42]
		Inhibiting angiogenesis	[45]
	Promoting cancer	Supporting biosynthesis through enhanced glutamine- dependent reductive carboxylation	[25]
**α-KG**	Inhibiting cancer	HIF-1α degradation and DNA methylation alterations	[59]
		Hypomethylation of DNA and histone H3K4me3	[60]
		Inducing pyroptosis through increasing ROS production and the cleavage of GSDMC by caspase-8	[83]
		Inducing ferroptosis by increased formation of ROS	[84,85]
		Inhibiting TGF-β and VEGF	[86]
		Mediating a dynamic conversion from glycolysis to OXPHOS	[88]
		Disrupting the NAD^+^/NADH balance and impeding OXPHOS	[89]
		Decreasing T_reg_ differentiation and increasing the generation of inflammatory cytokines	[93]
		Up-regulating PD-L1 and MHC-I expression and activating T cell	[99–101]
	Promoting cancer	Forming oncometabolite 2-HG in cancers with IDH mutations	[67]
		Activating mTORC1 and inhibiting autophagy	[80]
		Activating IKKβ and NF-κB and up-regulating GLUT1	[82]
		Augmenting macrophages M2 polarization and limiting M1 activation	[91,92]
	Complex effects	As an indispensable substrate of αKGDDs	[51]
**Succinate**	Inhibiting cancer	Inhibit the homologous recombination DNA repair pathway	[118]
	Promoting cancer	DNA hypermethylation	[114]
		Increasing expression of HIF-1α	[116,117]
		Activating STAT3 and ERK1/2 by SUCNR1, up-regulating VEGF expression, and promoting angiogenesis	[124]
		Increasing ROS production, activating NF-κB, and stabilizing HIF-1α	[126,127]
		Causing damage to p53	[129]
		Down-regulating KCNQ1 by activating serum/glucocorticoid- regulated kinase 1	[130]
		Promoting macrophage converting to TAMs and M2 polarization	[131]
		Inhibiting degranulation and expression of IFN-γ and TNF-α in T cells	[134]
		Impaired the cGAS/IFN-β pathway by SUCNR1, decreasing the secretion of CCL5 and CXCL10, and restraining the recruitment of CD8^+^ T cells	[135]
**Succinyl-CoA**	Complex effects	Inducing the posttranslational modification succinylation	[137–141]
**Fumarate**	Inhibiting cancer	Inhibit the homologous recombination DNA repair pathway	[118]
	Promoting cancer	DNA hypermethylation	[114]
		Repressing miR-200, activating EMT-associated transcription factor ZEB2, and eliciting EMT	[151]
		Stabilizing HIF-1α and promoting HIF-1α transcription	[153]
		Inducing the posttranslational modification succination	[156–162,171]
		Promoting aerobic glycolysis and reducing levels of AMPK and p53	[164]
		Participate in the biosynthesis and degradation of haem	[165]
		Using the inverse activity of ASL with arginine to generate argininosuccinate	[166]
		Relying on purine salvage for nucleotide biosynthesis	[167]
		Disrupting redox homeostasis	[168]
**Malate and OAA**	Promoting cancer	Increasing the NADPH/NADP^+^ ratio and maintaining redox homeostasis by malate–aspartate shuttle	[174]
	Inhibiting cancer	Inducing apoptosis and ROS accumulation by OAA supplementation	[177]
		Enhancing OXPHOS and inhibiting glycolysis by OAA supplementation	[177]

It is well known that rapidly proliferating cells, such as tumor cells, tend to obtain energy through glycolysis to support their rapid growth despite under aerobic conditions, which is the Warburg effect [5]. Warburg initially hypothesized that cancer cells or proliferating cells had mitochondrial defects that disrupt aerobic respiration and subsequently increase glycolytic metabolism [6]. Metabolic reprogramming is a hallmark of tumors, and substantial evidence indicates that tumor cells exhibit a switch from OXPHOS to glycolysis. Metabolic reprogramming enables tumor cells to survive, proliferate, and eventually outcompete normal cells. However, further research has revealed that the mitochondria of most cancer cells remain intact [7]. This suggests that the preference for glycolytic metabolism, despite its relative inefficiency in generating ATP, is driven by the fact that cancer cells have critical needs beyond energy production [8]. Cancer cells necessitate metabolic flexibility to support various cellular processes, such as nucleotide synthesis, macromolecule biosynthesis, and the maintenance of redox balance. Glycolysis facilitates rapid glucose uptake and provides metabolites for anabolic pathways, satisfying the high demand for essential building blocks for cell growth and division. Moreover, metabolic by-products like lactate generated in glycolysis can be utilized by neighboring cells within the tumor microenvironment (TME) [9].

In recent decades, it has been widely believed that tumor cells rely predominantly on aerobic glycolysis and suppress mitochondrial respiration. However, emerging evidence challenges this conception, indicating that not all tumors exhibit the characteristic metabolic phenotype of aerobic glycolysis. Several types of tumors still maintain functional mitochondria, including intact respiration processes. Moreover, some tumors even exhibit increased levels of OXPHOS [10]. Therefore, the TCA cycle has emerged as a central metabolic hub with profound implications for tumor growth. Beyond its role in energy production, the TCA cycle provides essential metabolites for cancer cell growth. TCA metabolites, including citrate, α-ketoglutarate (α-KG), succinate, and fumarate, play crucial roles in tumorigenesis and tumor progression. Citrate and α-KG can be affected by the glycolysis pathway or through the Warburg effect. Furthermore, succinate and fumarate have been characterized as oncometabolites, which also include 2-hydroxyglutarate (2-HG) [11]. In addition, excess lactate accumulation, which is commonly observed in the Warburg effect, contributes to TCA-cycle-associated metabolism. Interestingly, in certain tumors, lactate supersedes glucose as the preferred fuel source for the TCA cycle. This metabolic phenomenon underscores the versatility of cancer cells and their ability to adapt to various nutrient conditions [12].

This article presents a comprehensive review of the alterations observed in the TCA cycle metabolites in tumors, which affect tumor metabolism, signal transduction pathways, and even immune cells and cytokines within the TME.

## Citrate: A Double-Edged Sword

**Fig. 2.**
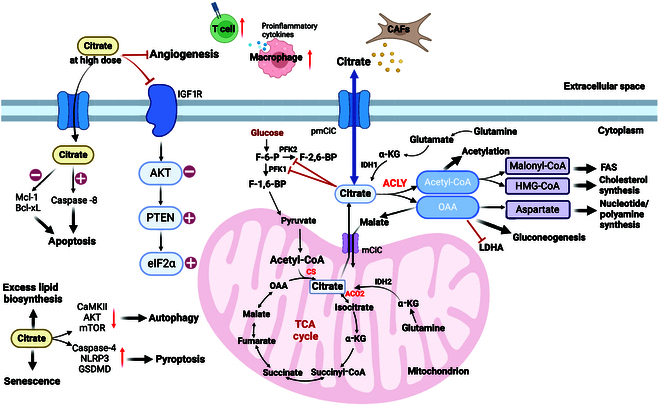
Citrate metabolism and signal transduction. In the mitochondria, citrate can be produced by CS-mediated condensation of OAA and acetyl-CoA and can also derive from glutamine. Citrate enters the cytoplasm via mCiC and antagonizes the glycolysis-related enzyme PFK1/2. Intracellular citrate can also be supplemented by glutamine. Citrate is cleaved to acetyl-CoA and OAA catalyzed by ACLY. Acetyl-CoA is involved in protein acetylation. In addition, acetyl-CoA can be converted to malonyl-CoA to participate in FAS or to HMG-CoA to enter cholesterol synthesis. OAA can be converted to aspartate to participate in nucleotide and polyamine synthesis, enter the gluconeogenesis process, or be converted to malate and returned to the mitochondrial. OAA competitively inhibits LDHA. Citrate can be transported to extracellular space by pmCiC, and CAFs in TME can also release citrate. Exogenous administration of high doses of citrate can activate caspase-8 and inhibit Mcl-1 and BCL-xL to induce apoptosis. Besides, citrate can activate autophagy and pyroptosis, inhibit angiogenesis, promote excess lipid biosynthesis, and induce cell senescence. Citrate binds to IGF1R, then down-regulates AKT, and up-regulates PTEN-eIF2α. High doses of citrate in TME can promote T cell infiltration and enhance the secretion of proinflammatory cytokines in macrophages.

In mitochondria, acetyl-CoA enters the TCA cycle to form citrate with oxaloacetate (OAA) via the condensation reaction, which is catalyzed by citrate synthase (CS). Subsequently, citrate is converted to cis-aconitate and then isocitrate via aconitase 2 (ACO2). In addition, citrate can also be transferred from mitochondria to the cytoplasm in exchange for malate [13], which is mediated by mitochondrial citrate carrier [mCiC; solute carrier family 25 member 1 (SLC25A1)]. In the cytoplasm, ATP citrate lyase (ACLY) then cleaved citrate into acetyl-CoA and OAA [14]. Acetyl-CoA is used for protein acetylation and lipid synthesis. Malonyl-CoA formed by carboxylation of acetyl-CoA participates in fatty acid synthesis (FAS) through the formation of long-chain fatty acids. In addition, acetyl-CoA can also enter the cholesterol synthesis pathway by forming 3-hydroxy-3-methylglutaryl (HMG)-CoA through the catalysis of various enzymes. Cytoplasmic OAA can be transformed into aspartate and then participates in nucleotide and polyamine synthesis and in gluconeogenesis as a raw material. It can also be converted to malate, which then returns to the mitochondrial cycle (known as citrate–malate shuttle) [13]. In the cytoplasm, glutamine becomes another source of citrate, as its derivative α-KG can be reductively carboxylated to form citrate (Fig. 2) [15].

### Intratumoral citrate and associated enzymes involved in metabolism

Although decreased citrate levels have been found in various tumors due to metabolic shifts induced by the Warburg effect [16], a study focusing on pediatric astrocytomas revealed that patients with aggressive tumors exhibited significantly higher levels of citrate than those with indolent tumors, suggesting that citrate concentrations could serve as a prognostic indicator for tumor aggressiveness in this specific subset of patients [17]. Alterations in many of the abovementioned citrate metabolic enzymes are also observed in tumors. Increased expression of CS was detected in some tumors, such as pancreatic cancer [18]; however, in other’s kind of tumors, such as cervical cancer, expression of CS showed a decreasing trend. Inhibition of CS caused epithelial–mesenchymal transition (EMT) and severe respiratory deficits, while tumor cells exhibited enhanced glycolysis. This alteration may be attributed to dysregulation of the p53/tumor protein p53-induced glycolysis and apoptosis regulator (TIGAR) and synthesis of cytochrome c oxidase 2 (SCO2) pathways, resulting in tumor metastasis and proliferation [19].

The expression of citrate carrier (CIC) was generally increased in breast cancer cells, and elevated levels of CIC indicated tumor metastasis and poor prognosis in breast cancers [20]. Up-regulated CIC levels were also observed in tumors including colorectal cancer, liver cancer, and lung cancer. Accordingly, CIC inhibitors limit the growth of different types of tumors. Besides, high expression of the plasma membrane citrate transporter (pmCiC; SLC13A5) correlates with aggressiveness of many human cancers [21].

In addition, ACLY is up-regulated in many tumors, and inhibiting ACLY can restrict tumor growth. An increase in ACLY in colon cancer supported the migration of cancer cells via stabilizing the CTNNB1 (β-catenin 1) protein [22]. In melanoma, increased ACLY expression promoted the acetyltransferase activity of P300 and enhanced the histone acetylation at the melanocyte-inducing transcription factor (MITF) locus. These epigenetic alterations specifically activated the MITF-PPARG coactivator 1 alpha (PGC1) axis, which facilitated OXPHOS and tumor growth. Furthermore, inhibition of ACLY suppressed the MITF-PGC1 axis, resulting in reversing the adaptive resistance to mitogen-activated protein kinase (MAPK) inhibition [23]. In pancreatic ductal adenocarcinoma, loss of ACLY blocked acinar-to-ductal metaplasia and tumorigenesis [24]. ACLY also had been documented in regulation of tumor immune microenvironment. Inactivation of the immune checkpoint receptor programmed cell death 1 (PD-1) on T cells enhances ACLY activity, and the subsequent increase in histone acetylation excessively activates the transcription factor activating protein-1. Pharmacological inhibition of ACLY blocks this process and suppresses the tumor. (Fig. 2).

Overall, the up-regulation of enzymes involved in the synthesis, transport, and catabolism of citrate has been observed in a variety of tumors. This up-regulation signifies the dependency of tumor cells on citrate and highlights the potential role of rapid citrate turnover in maintaining optimal intracellular citrate levels. Furthermore, this metabolic adaptation promotes the proliferation and metastasis of tumor cells.

### Citrate and tumor metabolic rewiring

In the well-functioning mitochondria of tumor cells, citrate is produced by the oxidation of glucose and glutamine-derived carbon. Tumor cells with mitochondrial defects convert glucose to lactate and primarily utilize glutamine-dependent reductive carboxylation for the formation of citrate [25]. In the cytoplasm, glutamine-derived α-KG undergoes reductive carboxylation to form citrate catalyzed by nicotinamide adenine dinucleotide phosphate (NADP)/reduced form of NADP^+^ (NADPH)-dependent isocitrate dehydrogenase 1 (IDH1), while IDH2 catalyzes the metabolic conversion in mitochondria. Subsequently, citrate provides acetyl-CoA for lipid synthesis and the synthesis of the remaining TCA cycle metabolites and related 4-carbon metabolites. This reductive glutamine-dependent pathway is the predominant metabolic mode in malignant cells that contain mutant complex I or complex III in the ETC or renal cancer cells from patients harboring fumarate hydratase (FH) mutations [26]. The production of α-KG can be sustained either through the MYC pathway or the serine pathway. In the serine pathway, 3-phosphohydroxypyruvate and glutamate are converted to 3-phosphoserine and α-KG by the enzyme phosphoserine aminotransferase (PSAT). This elucidates the role of PSAT as an important enzyme in the serine pathway for the transformation of substrates into α-KG [27]. Meanwhile, in breast cancer cells, the reduction of α-KG can be achieved by suppressing phosphoglycerate dehydrogenase (PHGDH), which catalyzes the initial step of the 3-step serine biosynthetic pathway. Conversely, in cells where PHGDH is overexpressed, the serine pathway contributes approximately 50% of the total anaplerotic flux of glutamine into the TCA cycle [28].

Citrate exerts a crucial influence on the glycolysis and gluconeogenesis pathways in cancer cells and serves as a potent allosteric activator of fructose-1,6-bisphosphatase (FBP) [29]. Furthermore, citrate acts as an allosteric inhibitor of phosphofructokinase 1 (PFK1) [30]. This dual regulation by citrate affects the balance between glycolysis and gluconeogenesis in tumor cells. FBP serves as a critical rate-limiting enzyme in the gluconeogenesis pathway and inhibiting FBP results in truncated gluconeogenesis. Furthermore, FBP promotes a shift from glycolysis to OXPHOS, which has a tumor-suppressive effect. FBP plays a role in regulating the Warburg effect in tumor cells by inhibiting hypoxia-inducible factor 1α (HIF-1α). This regulatory mechanism is influenced by citrate levels and highlights the intricate interplay between FBP, citrate, and the metabolic adaptations observed in tumor cells [31]. FBP has a essential impact on glucose uptake and the glycolytic flux by effectively suppressing HIF-1α and down-regulating its target genes, including GLUT1, hexokinase 2 (HK2), PFK1, and lactate dehydrogenase A (LDHA). Conversely, FBP enhances the flow of pyruvate into the TCA cycle, promoting OXPHOS and increasing oxygen consumption. This effect is achieved by blocking the expression of another HIF-1α-induced gene, pyruvate dehydrogenase kinase 1 (PDK1), which is associated with the inactivation of pyruvate dehydrogenase (PDH) [32].

PFK1 is responsible for a key step in glycolysis and produces fructose-1,6-bisphosphate (F1,6BP). F1,6BP facilitates the Warburg effect and tumor invasion through the activation of the inactive form of the pyruvate kinase M2 (PKM2) dimer and the up-regulation of RAS and downstream pathways including the MAPK and phosphatidylinositol 3-kinase (PI3K)/AKT pathways [33]. Meanwhile, F1,6BP is also an allosteric activator of pyruvate kinase (PK), which catalyzes the last step of glycolysis, suggesting that citrate can indirectly inhibit PK to suppress glycolysis [34]. Citrate also inhibits the glycolytic enzymes PFK2 to reduce the production of fructose-2,6-bisphosphate (F2,6BP), which acts as both an inhibitor of FBP and a vital activator of PFK1. In addition, citrate inhibits LDHA indirectly by increasing ACLY-mediated OAA production as an additional mechanism for inhibiting glycolysis. OAA shares structural similarity with 2 substrates of LDHA, pyruvate and α-KG, and is proved to be a competitive inhibitor of LDHA [35]. The increase in the Warburg effect in tumors decreases mitochondrial synthesis of citrate, while cancer cells have up-regulated genes such as ACLY to accelerate the transformation of citrate and maintain FAS and the biosynthesis of cholesterol, resulting in continuous consumption of citrate and reduced cytoplasmic citrate levels. As a result, the inhibitory effects of citrate on PFK1 and PFK2 and the activation of FBP are attenuated, which, in turn, affects gluconeogenesis and glycolysis [36].

Cancer cells require precise regulation of citrate concentrations to ensure their survival. They need an adequate supply of citrate to support the synthesis of essential molecules like fatty acids. However, excessive accumulation of citrate can disrupt glycolysis and hinder ATP production. Hence, maintaining a delicate balance in citrate levels is crucial for the metabolic needs of cancer cells. The control of citrate by cancer cells also presents potential therapeutic targets for cancer treatment. Modulating citrate level may provide novel approaches for therapeutic interventions (Fig. 2).

### The high citrate concentrations shows antitumor activity

Studies have demonstrated that administering exogenous sodium citrate or citrate can significantly increase intracellular citrate levels. Cancer cells with an increased demand for citrate exhibit a response to high levels of extracellular citrate. This uptake occurs through pmCiC from the extracellular space, including blood and cancer-associated cells [37]. The excessive elevation of citrate concentrations exerts potent inhibitory effects on the proliferation and growth of various types of tumor cells.

One mechanism of the tumor-suppressive effect of citrate is inducing multiple types of cell death pathways. Citrate has been documented to induce apoptosis via activating caspase-8 and caspase-2 and trigger the initiation of apoptosis by the activation of caspase-3, the cleavage of adenosine 5′-diphosphate (ADP)-ribose polymerase, and the release of cytochrome c [38]. It is well known that members of the BCL-2 family governed apoptosis and major antiapoptotic molecules involved are Bcl-2, Mcl-1, and BCL-xL. Citrate exhibit strong cytotoxic activity by effectively reducing the early expression of Mcl-1 and inducing apoptosis via the mitochondrial pathway in gastric cancer. Moreover, citrate’s ability to inhibit Mcl-1 expression can enhance the effectiveness of BCL-xL inhibitors on ovarian cancer cells [39]. In addition, citrate exerted anticancer effects by activating autophagy in prostate cancer cells through down-regulating the calcium- and calmodulin-dependent protein kinase II (CaMKII)/AKT/(mTOR) pathway [40]. Citrate also plays important roles in inducing pyroptosis and then effectively inhibiting tumor growth in ovarian cancer through activation of the caspase-4/NLR family pyrin domain containing 3 (NLRP3)/Gasdermin D (GSDMD) pathway [41].

Other possible mechanisms include excess lipid biosynthesis within tumor cells and the induction of cellular senescence, which involve the ataxia telangiectasia mutated (ATM)-associated DNA damage response, activation of the extracellular signal–regulated kinase 1/2 (ERK1/2) and p38 MAPK pathways, and mTOR kinase signaling [42]. Besides, citrate treatment altered the metabolism of tumor cells. Citrate modulated glycolysis in different tumor tissues through different targets (PFK1, aldolase, and phosphoglycerate kinase), activated eukaryotic translation initiation factor 2α (eIF2α) by down-regulating the insulin-like growth factor 1 receptor (IGF1R)/AKT pathway and up-regulating the phosphatase and tensin homolog (PTEN)/eIF2α pathway, and simultaneously impacted the TCA cycle to inhibit tumor growth [43]. Exogenous citrate supplement also induced the degradation of HIF-1α and suppressed hepatocellular carcinoma growth with hampered glycolysis in a hypoxic environment [44]. Moreover, citrate has shown promising potential as an antitumor substance by effectively inhibiting angiogenesis [45].

Interestingly, the administration of citrate could remodel TME by increasing the levels of tumor-infiltrating lymphocytes, specifically T lymphocytes in lung tumor tissues. Furthermore, citrate treatment enhances the secretion of proinflammatory cytokines in macrophages, contributing to an augmented immune response [43]. Moreover, sodium citrate, an alkaline salt, has the unique capability to neutralize the acidic environment of the TME, which is crucial for enhancing drug penetration and facilitating immune cell infiltration, thereby serving as a valuable auxiliary factor (Fig. 2) [46].

### The role of lower citrate concentrations in tumorigenesis

Cancer cells acquire citrate from the cancer-associated matrix and transport it into the cytoplasmic matrix by pmCiC to support cellular nutrients for a malignant phenotype [47]. In addition, citrate released by the cancer-associated matrix can induce tumor progression, specifically by fueling aggressiveness and organ colonization. The specific pmCiC inhibitor gluconate could be applied to suppress tumor growth, reduce stromal transformation and angiogenesis, and induce immune cell infiltration in vivo [21]. Notably, citrate-rich organs such as the liver, brain, and bone, are common targets of cancer cell metastasis and invasion, and tumors originated from these specific organs show minimal tendency of metastasis. Besides, cancer-associated fibroblasts (CAFs) release citrate via pmCiC, suggesting a considerable source of extracellular citrate in tumor-associated stroma [37] and indicating that citrate is a pivotal factor in the intricate communication between cancer cells and neighboring tissues. Its extracellular presence is critical for driving tumor metastasis (Fig. 2).

## α-KG: A Hub Connecting the TCA Cycle and Glutamine Metabolism

**Fig. 3.**
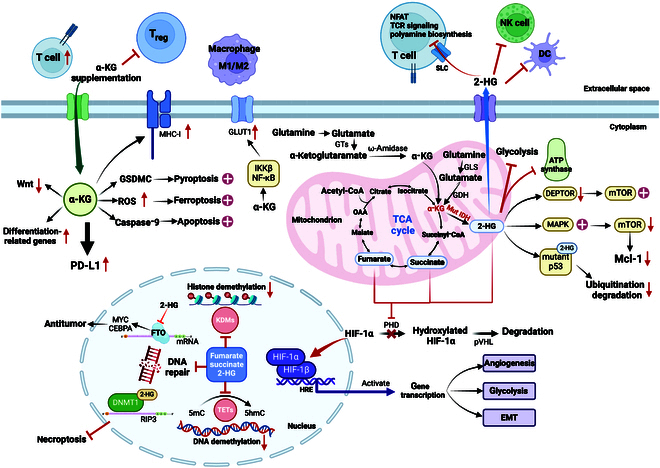
Overview of α-KG biosynthetic pathways and impact on tumor cells. α-KG can be formed either by isocitrate in the TCA cycle or by glutamine in the cytoplasm or mitochondria via different pathways. IDH mutation leads to the accumulation of 2-HG. As oncometabolites, 2-HG, succinate, and fumarate are proved to be competitive inhibitors of αKGDDs. Increased oncometabolites inhibit PHD and subsequent HIF-1α degradation. Overexpressed HIF-1α and downstream genes promote tumors by facilitating angiogenesis, glycolysis, and EMT. Oncometabolites can inhibit DNA and histone demethylases, meanwhile inhibiting DNA repair. D-2-HG binds to DNMT1 and induces hypermethylation of the RIP3 promoter to inhibit necroptosis. In addition, D-2-HG reduces DEPTOR protein stability and activates mTOR. D-2-HG activates AMPK and inhibits mTOR, resulting in the decrease of Mcl-1. 2-HG binds to mutant p53 to reduce its degradation. D-2-HG exerts antitumor effects by inhibiting FTO and then MYC/CEBPA signaling, along with inhibiting glycolysis and ATP synthase. D-2-HG suppresses the antitumor effects of T cells, NK cells, and DCs in TME. α-KG up-regulates GLUT1 by activating IKKβ and NF-κB. Exogenous addition of α-KG results in pyroptosis, ferroptosis,and apoptosis of tumor cells. Besides, α-KG inhibits the WNT pathway and increases the expression of PD-L1, MHC-1, and differentiation-related genes. α-KG can promote T cells, inhibit T_reg_, and regulate macrophage M1/M2-related polarization. HRE, HIF-responsive element; NFAT, nuclear factor of activated T cells.

IDH catalyzed the oxidative decarboxylation of isocitrate to α-KG (2-oxoglutarate), which is irreversible. α-KG is then decarboxylated to form succinyl-CoA by the α-KG dehydrogenase (α-KGDH) complex in another irreversible reaction of the TCA cycle. Both processes use NAD^+^ as a cofactor. Glutamine is also an indispensable source of α-KG both in the cytoplasm and mitochondria. Glutamine is catalyzed by glutaminase (GLS) to generate glutamate and ammonia, and subsequent conversion of glutamate into α-KG is catalyzed by glutamate dehydrogenase (GDH). This process is called glutaminolysis [48]. Another pathway is coupled to glutamine transaminase (GT) and ω-amidase, in which GT catalyzes the formation of α-ketoglutaramate from glutamine. α-Ketoglutaramate is then hydrolyzed to form α-KG via ω-amidase. α-KG can also be directly derived from glutamate transamination by glutamate pyruvate transaminase and glutamate OAA transaminase (GOT) (Fig. 3).

### α-KG-dependent dioxygenases (2-oxoglutarate-dependent dioxygenases)

α-KG-dependent dioxygenase (αKGDDs) are a superfamily of enzymes that utilize α-KG and oxygen as substrates and iron and ascorbate as cofactors to generate succinate and carbon dioxide. αKGDDs require oxygen, reduced iron, and α-KG to function. However, ascorbate is not a direct substrate and prevents unintentional iron oxidation in uncoupled reactions [49]. αKGDDs can be classified into several broad categories: protein hydroxylases, histone demethylases, nucleic acid oxygenases, fatty acid and small-molecule oxygenases, and enzymes with unassigned catalytic functions [50]. These factors participate in multiple biological processes, containing HIF-mediated adaptation to hypoxia, extracellular matrix formation, DNA methylation, histone methylation, RNA processing, and protein translation [51]. αKGDDs is a link between cancer metabolism and epigenetics, and alterations in their function have vital implications in different tumor types. Several αKGDDs that play important roles in tumors are listed below.

HIF-1α, a member of the HIF transcription factor family, is a main regulator under hypoxia condition. Under normoxic conditions, prolyl hydroxylase (PHD) (belonging to the Egln family) hydroxylates the HIF-1α subunit, and then the hydroxylated subunit is degraded through the ubiquitin-proteasome pathway mediated by the von Hippel–Lindau tumor suppressor gene product (pVHL) [52]. PHD also belongs to αKGDD. Under hypoxic conditions, HIF-1α translocates into the nucleus to bind to HIF-1β rather than degradation and activate the transcription of downstream genes. Increasing evidence manifested that overexpression of HIF-1α and downstream genes promote tumor progression via the promotion of angiogenesis, glycolysis, and EMT [53].

Ten–eleven translocation (TET) as another αKGDD is involved in DNA demethylation. TET can mediate the partial conversion of 5-methylcytosine (5mC) to 5-hydroxymethylcytosine (5-hmC). TET can also produce 5-formylcytosine (5fC) and 5-carboxylcytosine (5caC) through oxidating 5mC in an enzyme-dependent manner. Thymidine DNA glycosylase specifically recognizes and excises 5caC and 5fC, which are replaced with unmodified cytosine, constituting the pathway of active DNA demethylation [54]. Another pathway depends on TET-mediated hydroxymethylation of a methylated CG site in vivo, which eliminates 5mC and impedes the maintenance of DNA methylation patterns [55]. Mutations of TET genes or reduced level of TET protein expression are not limited to malignant hematopoiesis leading to hematological tumors but have also been observed in many human solid tumors [56].

Another large category is histone demethylases. The methylation of lysine residues on histones is performed by histone lysine methyltransferases, while the removal of lysine methylation is dependent on lysine demethylases (KDMs), which include the FAD-dependent amine oxidase and Jumonji C-domain-containing demethylases (JMJD) [57]. Altered JMJD expression and its regulation of related genes may be a potential mechanism of tumorigenesis and tumor progression [58].

Extensive studies exhibited the multifaceted roles of αKGDD in tumors. Apart from directly targeting αKGDD itself, the αKGDD activity had been intervened by modulating the levels of its substrate, α-KG, to intervene in tumor development. This approach allows for a more targeted and precise therapeutic strategy for combating tumor progression. It has been shown that the knockdown of branched-chain amino acid transaminase 1 induces the accumulation of intracellular α-KG, which subsequently activates Egl-9 family hypoxia-inducible factor 1 (EGLN1) and TET enzymes. This activation triggers the degradation of HIF-1α and leads to alterations in DNA methylation patterns. Consequently, these molecular changes result in defective growth and impaired survival of cancer cells [59]. Exogenous supplementation of α-KG to tumors triggers a response from αKGDDs. This response induces DNA hypomethylation and histone H3 lysine-4 trimethylation (H3K4me3) modifications, resulting in the up-regulation of genes associated with cancer cell differentiation and the down-regulation of Wnt target genes, which remarkablely inhibit tumor growth [60]. Modulation of αKGDDs by α-KG has yielded effective antitumor effects in some tumors. However, the understanding of the roles of complex αKGDDs in cancer is still far from complete, and the overall intracellular effects induced by α-KG may not always be consistent. Therefore, it is necessary to advance the exploration about αKGDDs for promising antitumor strategies.

### IDH and tumors

The IDH family consists of 3 isozymes, of which IDH2 and IDH3 locate in the mitochondria, while IDH1 locates in the cytoplasm and peroxisomes. IDH1 is involved in amino acid utilization and the metabolism of glucose and lipids. IDH1 can catalyze isocitrate dehydrogenation to produce α-KG, simultaneously converting a molecule of NADP^+^ to NADPH. By generating NADPH, IDH1 also promotes the conversion of glutathione (GSH) disulfide to GSH, which functions as an antioxidant scavenging reactive oxygen species (ROS) [61]. IDH2 regulates the TCA cycle and prevents oxidative stress, while IDH3 is a crucial enzyme in the TCA cycle to perform oxidative decarboxylation.

IDH1/2 are frequently mutated in glioblastoma [62], acute myeloid leukemia (AML) [63], thyroid carcinoma [64], and chondrosarcoma [65]. In gliomas, the maximum frequency of IDH1 mutation is greater than 90%, while the frequency of IDH2 is less than 5% [66]. IDH1 and IDH2 mutations lead to NADPH-dependent reduction of α-KG to form oncometabolite D-2-HG (R-2-HG), allowing intracellular D-2-HG accumulation to supraphysiological levels. Increased D-2-HG exerts oncogenic effects through multiple pathways, such as inhibiting αKGDDs, breaking the DNA repair, and inducing cell death.

D-2-HG is structurally semblable to α-KG, apart from the C2 hydroxyl group of D-2-HG replacing the C2 carbonyl group of α-KG [67]. Therefore, D-2-HG acts as a competitive inhibitor of αKGDDs. It represented the activity to the detriment of epigenetics and hypoxic regulation, by restraining αKGDDs such as the JMJD family and TET family [68]. This epigenetic dysregulation can induce genome-wide histone and DNA hypermethylation and promote oncogenesis. D-2-HG-induced KDM4A inhibition reduces DEP-domain-containing mTOR-interacting protein (DEPTOR) stability. DEPTOR is a negative regulator of mTORC1/2, and inhibiting DEPTOR allows activation of the mTOR signaling pathway [69].

In addition, pathological concentrations of 2-HG in cancers can markedly inhibit AlkB homolog 2/3, the enzymes related to human DNA repair. The DNA repair process is inhibited, and the increased mutation rate exacerbates tumorigenesis [70].

2-HG also has effects on apoptosis and necrosis. Energy depletion induced by 2-HG promotes the activation of adenosine 5′-monophosphate kinase (AMPK), resulting in decreased mTOR signaling, which ultimately leads to decreased levels of the antiapoptotic protein Mcl-1. Inhibiting Bcl-xL results in the lethality of IDH mutant cells [71]. 2-HG also directly binds to mutant p53, thereby reducing ubiquitination and degradation of mutant p53, which is proved to inhibit apoptosis of tumor cells [72]. In addition, 2-HG combines with DNA methyltransferase 1 (DNMT1) and mediates receptor interacting serine/threonine kinase 3 (RIP3) promoter hypermethylation, thereby impairing RIP3-dependent necroptosis [73]. The impairment of necroptosis also contributes to IDH1/2-mutation-driven tumorigenesis.

Paradoxically, glioma and glioblastoma patients with IDH mutations have significantly longer overall survival than those patients with IDH wild type, and the same trend has been observed in patients with AML [74]. Studies have shown that in leukemia and glioma, 2-HG exerts antitumor effects by inhibiting fat mass and obesity-associated protein (FTO) involving in mRNA modification and downstream MYC/CCAAT enhancer binding protein alpha signaling [75]. In addition, 2-HG exerts antitumor effects by altering cellular metabolism. 2-HG reduced the growth and viability of IDH1-mutant glioma cells by binding to and inhibiting ATP synthase and downstream mTOR signaling. Furthermore, IDH1 mutant cells exhibit reduced ATP levels and mitochondrial respiration, which causes growth arrest and cytotoxicity when restricting glucose [76]. 2-HG primarily inhibits aerobic glycolysis in leukemia cells. This effect is achieved by targeting FTO/*N*^6^-methyladenosine/YTH *N*^6^-methyladenosine RNA binding protein F2 signaling. 2-HG then down-regulates the vital glycolytic genes PFKP and LDHB to exert the antitumor activity (Fig. 3) [77].

The paradoxical roles of 2-HG or IDH mutations are conceivable to be associated with different types or stages of tumors. The data show that IDH mutations occur in approximately 79% of low-grade primary gliomas but less than 10% of high-grade glioblastomas (GBMs) [78]. This suggests that the tumor suppression effect of 2-HG perhaps limits the further rapid progression of initiated gliomas. It also provides new options for unique treatment for different tumors.

### Metabolic remodeling in cancers mediated by α-KG

The metabolic reprogramming of tumor cells stimulates anabolism, resulting in cancer cells being highly addicted on glutamine [79]. Tumor cells produce α-KG in mitochondria via the enhanced glutaminolysis pathway and then activate mTORC1 [80]. The activation of mTORC1 inhibits autophagy and accelerates growth of tumor cells. α-KG can also be exported to the cytoplasm and carboxylated to form citrate catalyzed by IDH1 for FAS [81]. The α-KG produced by GDH directly activates inhibitor of nuclear factor κB (NF-κB) kinase β (IKKβ) and NF-κB signaling. Gliogenesis is then accelerated by the up-regulation of GLUT1 to facilitate the uptake of glucose and subsequent survival of cancer cells (Fig. 3) [82].

### The tumor-suppressing effect of α-KG

The addition of α-KG to tumors has shown promising results in antitumor therapies by inducing multiple types of death in tumor cells. Pyroptosis is one mechanism. Under circumstance of increased ROS and an acidic environment, α-KG induces the cleavage of gasdermin C (GSDMC) by death receptor 6-activated caspase-8, leading to pyroptosis [83]. Ferroptosis can also be promoted by α-KG-related metabolic processes. During this process, GLS2 promotes the conversion of glutamate to α-KG, inducing increased formation of ROS and ferroptosis [84]. ROS is also produced by lymphoma cells during the catalytic transformation of accumulated α-KG to 2-HG by malate dehydrogenase 1 (MDH1), which promotes ferroptosis by mediating lipid peroxidation and DNA-damage-related tumor protein p53 (TP53) expression [85]. Besides, α-KG induces apoptosis through c-Jun N-terminal protein kinase and caspase-9-dependent mechanisms, inhibits transforming growth factor-β (TGF-β) and vascular endothelial growth factor (VEGF), and exerts antiosteosarcoma effects in vitro [86]. α-KG also exerts tumor suppressive effects as an effector molecule of p53. Supporting the accumulation of α-KG can antagonize the malignant progression of p53-deficient tumors (Fig. 3) [87].

In breast cancer, the addition of α-KG mediates a dynamic conversion from glycolysis to OXPHOS [88]. Besides, α-KG supplementation induces metabolic synthetic lethality and synergistic inhibition of branched-chain amino acid transaminase 1 in GBM. This limits substrate catabolism by disrupting the NAD^+^/NADH balance and impedes OXPHOS, resulting in the inhibition of mTORC1 and a reduction in nucleotide biosynthesis [89].

### Effects on immune cells

Beyond its direct regulation of tumor cells fate, α-KG intricately manipulates the metabolic and epigenetic reprogramming of immune cells within the TME, thereby affecting tumor development.

α-KG provides support for macrophage activation and phenotypic changes. In M1 macrophages, the TCA cycle is blocked after citrate and after succinate, allowing for the accumulation of citrate and succinate. In contrast, M2 macrophages have an intact TCA cycle and can exclusively produce metabolites that promote protein glycosylation. Therefore, M1 macrophages use Warburg metabolism, whereas M2 macrophages are dedicated to OXPHOS [90]. α-KG from glutamine can activate M2 macrophages through JMJD3-dependent epigenetic reprogramming and fatty acid oxidation regulation, while regulating IKKβ activity through PHD-mediated inhibition of NF-κB pathway, thereby limiting M1 activation [91]. The accumulation of α-KG is regulated by SUMO-specific peptidase 1/ sirtuin 3 signaling during macrophage M2 polarization. Activation of this axis can enhance glutaminolysis through the deacetylation of GDH1, leading to the accumulation of α-KG to augment M2 polarization [92].

Studies have shown that an increase in OXPHOS induced by increased α-KG significantly decreases regulatory T cell (T_reg_) differentiation and increases the generation of inflammatory cytokines [93]. It is associated with mitochondrial metabolism and lipidome remodeling. Adoptive transfer of T_regs_ treated with α-KG to tumor-bearing mice can maintain the inflammatory environment in vivo and limit tumor growth. Moreover, the production of 2-HG specifically alter the epigenetic state of CD4^+^ T cells by altering the methylation level of the forkhead box p3 gene locus, which disrupts the balance between T helper 17 cells and inducible T_regs_ [94]. Decreased expression of cytotoxic T-lymphocyte-related genes and interferon-γ (IFN-γ)-induced chemokines, such as C-X-C motif chemokine ligand 10 (CXCL10), suppressed T cell targeting to tumor sites, which was associated with reduced signal transducers and activators of transcription 1 (STAT1) production in IDH-mutated tumors [95]. T cells import 2-HG secreted by tumor cells via a specific SLC transport system. Then, 2-HG disturbs the transcription of nuclear factor of activated T cells, ATP-dependent T cell receptor (TCR) signaling and polyamine biosynthesis, resulting in impaired T cell activation and antitumor immunity [96]. 2-HG can also inactivate of natural killer (NK) cells and dendritic cells (DCs) in TME and participate in the immune evasion of tumors [97].

α-KG is also involved in the regulation of programmed cell death 1 ligand 1 (PD-L1) and major histocompatibility complex I (MHC-I) expression. α-KG was shown to activate IFN-γ-induced p-STAT1 and p-STAT3 [98]. Furthermore, the observable increase in TET2/3 elevates the 5-hmC level in the PD-L1 promoter, and the combination of STAT1/3 and the PD-L1 promoter was stabilized, thus up-regulating IFNG–STAT1/3–PD-L1 signal transduction [99]. In renal cell carcinoma, α-KG up-regulates β_2_-microglobulin by weakening the enrichment of H3K4me1 in the promoter region and promoting concomitant demethylation of H3K4me1. Subsequently, tumor growth is limited by up-regulating MHC-I expression and increasing CD8^+^ T cell infiltration and cytotoxicity [100]. Through increasing PD-L1 expression and activating T cell, α-KG in combination with anti-PD-1/PD-L1 immunotherapy showed considerable efficacy in various tumor models (Fig. 3) [101].

## Succinate: An Enhancer of Tumorigenesis through Modulating the TME

The transformation of succinyl-CoA to succinate is catalyzed by succinyl-CoA synthase, which is accompanied by substrate-level phosphorylation to generate ATP. Succinate is dehydrogenated to produce fumarate catalyzed by succinate dehydrogenase (SDH) with the coenzyme FAD. SDH is also the mitochondrial complex II and succinate-ubiquinone oxidoreductase in ETC.

### Succinate and SDH

SDH consists of 4 mitochondrial subunit proteins (SDHA, SDHB, SDHC, and SDHD) and 2 auxiliary subunits (SDHAF1 and SDHAF2). SDHA and SDHB compose the catalytic component, whereas SDHC and SDHD are the anchoring components attaching the SDH complex to the inner mitochondrial membrane. Deleterious mutations in any subunits can abrogate the function of the entire enzyme complex by releasing SDHB into the cytoplasm and promoting rapid SDHB degradation [102].

Mutations in SDHx subunits are associated with many tumors, including familial paraganglioma (PGL) [103], pheochromocytoma (PHEO) [104], gastric stromal tumors [105], renal cell carcinoma [106], pituitary adenoma [107], papillary thyroid carcinoma [108], pancreatic neuroendocrine tumor [109], and gastric and colorectal carcinoma [110]. There are increasing reports of the coexistence of PHEO/PGL and pituitary adenomas, which are referred to as 3PAS.

SDH epigenetic alterations have also become one of the mechanisms of its altered activity. One study showed SDHC promoter methylation in PGL, suggesting a novel potential pathogenic mechanism [111]. MYC promotes the acetylation of lysine-335 of SDHA by stimulating the degradation of SIRT3 deacetylase mediated by s-phase kinase-associated protein 2. Consequently, inactivated SDHA triggered the activation of H3K4me3 and tumor-specific gene expression, which contributed to tumor growth. TNF receptor-associated protein 1, a mitochondrial chaperone, is highly expressed in many tumors. It can bind to SDH and inhibit its activity to promote tumor growth [112]. The mutation and deficiency of SDH cause abnormal accumulation of succinate. In addition, it has been shown that PTEN deficiency is related to increased accumulation of succinate and inhibiting the succinate plasma membrane transporter sodium-dependent dicarboxylate transporter member 3 alone cannot overcome it [113].

### Succinate: An inhibitor of αKGDDs and DNA repair

As structural α-KG analogs, succinate and its product fumarate act as α-KG competitors and broadly inhibit the activity of αKGDDs, including the JMJD family and the TET family. Succinate and fumarate alter the overall DNA methylation pattern, resulting in dramatic DNA hypermethylation [114]. The observed alterations in gene expression have a profound impact on various genes involved in cell differentiation and malignant properties, thereby playing a noteworthy role in driving tumorigenesis. Succinate also can enhance EMT and cancer cell stemness through epigenetic reprogramming [115].

Increased expression of HIF-1α was detected in SDHD-mutated hereditary PGL [116]. In ovarian cancer, SDHB-silenced cells up-regulated HIF-1α, and the p-AMPKα was activated [117]. Succinate accumulates in mitochondria in response to SDH inhibition and then translocates to the cytoplasm. An increase in succinate inhibits PHD and the hydroxylation of HIF-1α in the cytoplasm, and pVHL binding to HIF-α is decreased, leading to the stabilization of HIF-1α. This phenomenon is defined as “pseudo-hypoxia” and promotes tumor growth.

**Fig. 4.**
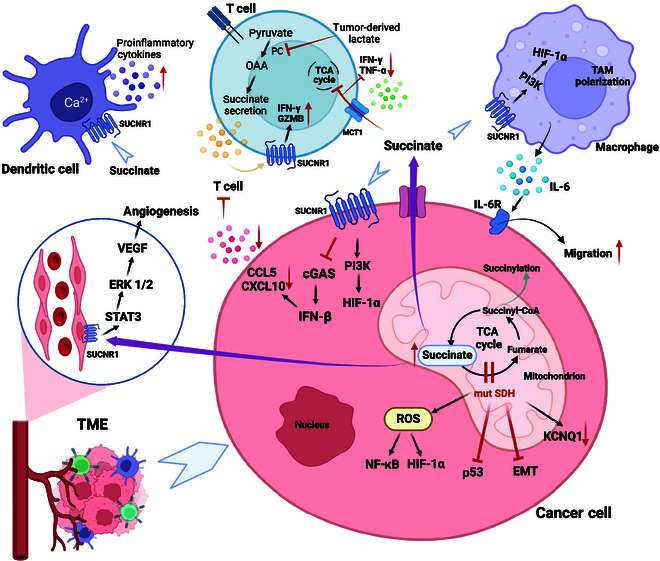
The role of succinate accumulation in tumors. SDH mutations lead to the truncation of TCA cycle, resulting in the accumulation of succinate. Subsequently, the increasing ROS activates NF-κB signaling and induces nuclear HIF-1α stabilization. In addition, SDH mutations also suppress p53 and promote EMT. The accumulation of succinate causes the down-regulation of KCNQ1 and promotes tumor progression. Extracellular succinate derived from tumor cells activates PI3K and HIF-1α by interacting with SUCNR1 on the surface of cancer cells, while inhibiting cGAS, resulting in decreased secretion of CCL5 and CXCL10 and inhibited recruitment of T cells. Succinate can also promote macrophage polarization to TAM and migration through the SUCNR1/PI3K/HIF-1α signaling pathway. TAM promotes the migration of cancer cells by secreting IL-6. Succinate inhibits the TCA cycle of T cells and the expression of cytokines such as IFN-γ and TNF-α. Tumor-derived lactate also inhibits the production of cytotoxic molecules such as IFN-γ and Granzyme B (GZMB) by affecting succinate secretion of T cells. Succinate promotes angiogenesis by activating the STAT3/ERK/VEGF pathway in endothelial cells through SUCNR1. Succinate acts on SUCNR1 on DC, resulting in enhanced DC migration and increased secretions of proinflammatory factors. Succinyl-CoA participates in the succinylation of lysine residues of proteins.

Recent studies have established a link between oncometabolites and DNA repair, which can be affected by many factors. High levels of succinate and fumarate inhibit the homologous recombination DNA repair pathway and induce an increase in DNA double-strand breaks through the inhibition of KDM4A and KDM4B, leading to enhanced sensitivity to poly(ADP-ribose) polymerase (PARP) inhibitors [118]. Investigation of how oncometabolites disrupt DNA repair revealed that oncometabolites inhibited KDM4B, resulting in abnormal hypermethylation of H3K9 at sites surrounding DNA breaks and masking local H3K9me3 signals. As a result, the recruitment of the critical proximal homology-dependent repair (HDR) factors, lysine acetyltransferase 5 and ATM, was severely impaired in response to DNA breakage, and there were decreased end excision and reduced recruitment of downstream repair factors, thereby affecting the execution of HDR and sensitivity to PARP inhibitors [119]. However, another study showed the opposite result. Following ionizing radiation, local accumulation of fumarate inhibited KDM2B histone demethylase, leading to increased H3K36me2, thereby increasing nonhomologous end joining DNA repair and cell survival (Fig. 4) [120].

### Succinate and succinate receptor 1

Succinate is a ligand for succinate receptor 1 (SUCNR1), which is likewise termed as G-protein-coupled receptor-91 (GPR91), and their interaction affects tumorigenesis. SUCNR1 overexpression has been found in various tumor types including renal cell carcinoma [121] and lung cancer [122]. Succinate promotes the proliferation of PHEO cells via SUCNR1 [123]. In addition, succinate can activate STAT3 and ERK1/2 by binding to SUCNR1, resulting in the up-regulation of VEGF expression and angiogenesis [124]. Besides, the presence of glutamine induces the up-regulation of SUCNR1, which is essential for glutamine-addicted tumor cells. Knocking down SUCNR1 in cancer cell lines significantly increased mitochondrial respiration, ROS production, and TCA cycle throughput, facilitating cancer cell lethality. SUCNR1 knockdown combined with the chemotherapy drugs cisplatin and gemcitabine ulteriorly accelerated cancer cell death (Fig. 4) [125].

The roles of SUCNR1 and its downstream signaling pathways in tumors are poorly understood and require further exploration. Available evidence suggests that the combination of succinate and SUCNR1 drives cancer metastasis and progression, underscoring its potential as a plausible target for tumor therapeutic agents.

### Other succinate signal pathways

The attenuation of SDH activity induced the increase in ROS levels. Subsequently, the activation of NF-κB signaling [126] or the stabilization of nuclear HIF-1α [127] promoted tumor growth and metastasis.

Furthermore, tumor cells exposed to succinate and fumarate in relatively high concentrations tend to undergo apoptosis [128]. Conversely, studies have found that SDHx mutations can cause damage to p53, which is a tumor suppressor gene mediating programmed cell death including apoptosis [129]. This contradiction may be due to the difference in the concentration of exogenously added metabolites and the intracellular metabolites.

SDH also seems to be regulated by hormones. A study of endometrial cancer showed that negative regulation of estrogen leads to decreased SDHB due to a decrease in the expression of ubiquitin C. As a result, the accumulation of succinate can down-regulate the expression of potassium-voltage-gated channel subfamily Q member 1 (KCNQ1) by the activation of serum/glucocorticoid regulated kinase 1, thereby promoting tumor progression (Fig. 4) [130].

### Succinate and immune cells

Tumor cells secrete succinate into the extracellular space, and macrophages with high expression of SUCNR1 sense succinate, thereby promoting their migration, conversion to tumor-associated macrophages (TAMs), and polarization. Polarized macrophages enhance the migration of cancer cells by secreting migration-promoting cytokines such as interleukin-6 (IL-6) and inducing tumor metastasis through PI3K/AKT and HIF-1α signaling [122]. Extracellular succinate can also activate the transcription of immunological genes in M2 macrophages through SUCNR1, forming a hyperpolarized M2 macrophage environment [131]. Not only can succinate affect macrophages, but macrophages can also affect succinate. In breast cancer, TAMs expedite tumor development by inhibiting SDH in tumor cells. Macrophage-dependent SDHD changes are mediated by the STAT and TGF-β pathways. Furthermore, breast cancer cells cocultured with proinflammatory M1 macrophages exhibited a decreased HIF-1a level, whereas the coculture with anti-inflammatory macrophages stabilized HIF-1α [132].

Cytotoxic CD8^+^ T cells depend on pyruvate carboxylase (PC) to replenish metabolites to maintain the TCA cycle, and this enzyme converts pyruvate directly into OAA. This enzyme also causes the secretion of succinate, which initiates autocrine signaling via SUCNR1. T cells then augment the generation of cytotoxic molecules including IFN-γ and Granzyme B to kill tumor cells. However, in the TME, tumor-derived lactate reverts this process to the traditional TCA cycle, in which pyruvate enters the TCA cycle by PDH, and succinate generates fumarate via the cycle rather than being secreted. Studies have shown that direct activation of SUCNR1 by agonists or by inhibiting PDH and increasing PC activity can overcome the inhibition of T cells and reactivate CD8^+^ T cells [133]. Besides, when exposed to tumor-associated concentrations of succinate, T cells down-regulated SUCNR1 after being activated and took up extracellular succinate partially through solute carrier family 16 member 1 (MCT1), leading to significant inhibition of their degranulation and expression of IFN-γ and tumor necrosis factor-α (TNF-α). Suppression of T cell function depends on the inhibition of glucose flux through the TCA cycle, along with the SUCNR1 signaling pathway. Cancer genome sequencing of patients with SDH mutations revealed severe repression of IFN-γ-induced genes [134]. In addition, the study on colorectal cancer showed that an increase in serum succinate levels caused by gut microbial metabolism impaired the cGAS/IFN-β pathway by binding to SUCNR1, which is a receptor on tumor cells. Subsequently, the secretion of the chemokines C-C motif chemokine ligand 5 (CCL5) and CXCL10 was decreased, restraining the recruitment of CD8^+^ T cells and directly reducing the response to anti-PD-1 immunotherapy [135].

Dendritic cells have high GPR91 expression. In DCs, succinate triggers intracellular calcium mobilization and migratory responses through GPR91, functioning as an extracellular mediator. Succinate also synergizes with the ligands of Toll-like receptors (TLRs) to produce proinflammatory cytokines and enhance immune response (Fig. 4) [136].

### Succinylation

Succinylation of lysine is a newly discovered posttranslational modification. In the process, succinyl groups are transferred from succinyl-CoA to the lysine residues of proteins, through enzymatic or nonenzymatic methods. Succinyl-CoA can be derived from the TCA cycle and the metabolism of lipid and various amino acids. Succinylation was first discovered in *Escherichia coli* and later in eukaryotes. Succinylation occurs mainly in mitochondria and more than one-third of nucleosomes as well as histones and nonhistones in the nucleus. The impacts of succinylation on tumorigenesis and tumor development are widely studied.

Lysine succinylation of chromatin proteins is common and increases in response to the loss of SDH [137]. Histone modifications are the center of the processes. The α-KGDH complex localizes in the nucleus and binds to lysine acetyltransferase 2A (KAT2A) in the gene promoter region that has succinyltransferase activity. This binding results in the local production of succinyl-CoA and then the histone H3 succinylation, triggering gene transcription and tumor cell proliferation. Preventing α-KGDH complex from entering the nucleus or decreasing KAT2A expression can inhibit tumor growth [138].

Protein succinylation in tumors has also been extensively studied. Carnitine palmitoyltransferase 1A (CPT1A) is capable of using succinyl-CoA as a substrate and exhibits lysine succinyltransferase activity to regulate of cellular metabolism [139]. Studies have shown that CPT1A succinylates K222 on LDHA, restricting the combination of LDHA and sequestosome 1 and subsequent lysosomal degradation of LDHA, thereby promoting the invasion and growth of gastric cancer [140]. Another study found that GLS succinylation enhanced by succinate-CoA ligase ADP-forming subunit beta (SUCLA2) phosphorylation facilitated tumor growth by counteracting oxidative stress [141].

Succinylation can also be negatively regulated by SIRT5 and SIRT7. The desuccinylase SIRT5 mediates the desuccinylation of lysine-280 on serine hydroxymethyltransferase (SHMT2), which, in turn, drives cancer cells proliferation and adaptation to serine metabolism for rapid growth [142]. SIRT5 also inhibits SDH activity through the desuccinylation of SDHA [143]. SIRT7 was also proved to be a NAD^+^-dependent histone desuccinylase, particularly upon DNA damage. SIRT7 is recruited to DNA double-strand breaks in a PARP1-dependent manner and catalyzes the desuccinylation of H3K122, promoting chromatin condensation and double-strand break repair [144,145].

## Fumarate: An Epigenetic Modifier

Fumarate is formed by the dehydrogenation of succinate, and then the reversible hydration of fumarate is catalyzed by FH to form malate. In the ETC, fumarate is also the terminal electron acceptor, which is originally oxygen. When oxygen reduction is impaired, cells enable electron deposition onto fumarate by driving the SDH complex, and then fumarate reduction sustains the activities of dihydroorotate dehydrogenase and complex I [146].

### Fumarate and FH

Heterozygous germline mutations in FH predispose patients to hereditary leiomyomatosis and renal cell cancer (HLRCC), which is a cancer syndrome characterized by uterine fibroids, skin leiomyomata, and aggressive papillary renal cell cancer [147]. In addition, FH deficiency confers susceptibility to malignant PGL and PHEO [148]. Other rare FH-deficient tumors include testis Leydig cell tumors, breast cancer, and bladder cancer [149]. FH deficiency results in the truncation of the TCA cycle and fumarate accumulation up to the millimolar levels. Fumarate accumulation can also be caused by the up-regulation of mTOR [150]. High levels of fumarate can translocate from mitochondria to cytoplasm and nucleus and can also be secreted to extracellular space, affecting the occurrence and development of tumors.

### Fumarate and αKGDDs

As mentioned previously, fumarate, which is a competitive inhibitor of αKGDDs, could accumulate to repress miR-200 through TET-mediated epigenetic alterations, leading to the expression of EMT-associated transcription factor zinc finger E-box binding homeobox 2 [151]. When the expression of FH is decreased via the chromatin remodeling mediated by lymphoid-specific helicase, it drives tumor progression through eliciting EMT. This could serve as evidence of the association between fumarate and EMT [152]. Fumarate can also competitively inhibit PHDs and stabilize HIF-1α, thereby activating target genes. In addition to this canonical pathway, fumarate was shown to promote the phosphorylation and accumulation of p65 on the HIF-1α promoter through Tank-binding kinase 1 (TBK1). This promoted HIF-1α transcription through IKK-independent noncanonical NF-κB signaling [153].

### Succination

S-(2-succinyl) cysteine (2SC) is formed by a Michael addition reaction between fumarate and the cysteine sulfonyl group in the protein and is stabilized chemical modification of the cysteine residue. This process is described as the succination, which is different from succinylation, and many proteins are identified as targets of succination [154]. This posttranslational modification is irreversible and resistant to acid hydrolysis, occurring in the presence of pathological levels of fumarate. Inactivated FH results in high levels of protein succination. Studies have shown that 2SC-modified proteins, which correctly predict genetic alterations in FH, are specific and sensitive metabolic biomarkers of HLRCC [155].

**Fig. 5.**
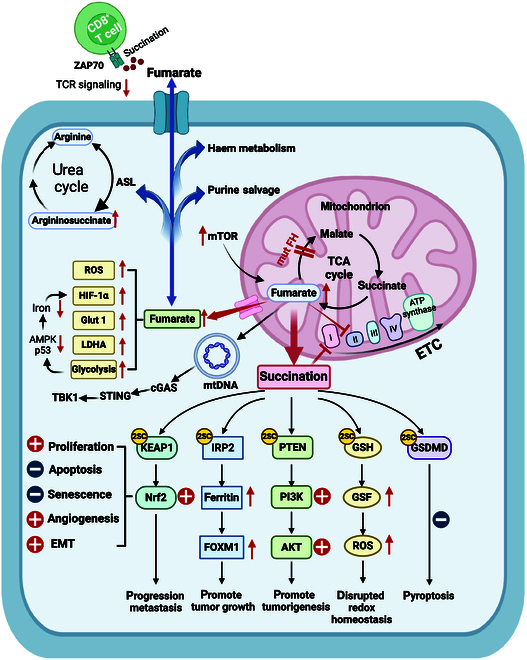
The role of fumarate accumulation in tumors. FH mutations in some tumors lead to fumarate accumulation, which can also be caused by up-regulation of mTOR. FH mutant tumors are characterized by increased glycolysis and ROS, accompany by overexpression of HIF-1α, Glut1, and LDHA. Increased glycolysis induces decreased levels of AMPK and p53, resulting in cytoplasmic iron deficiency and increased HIF-1α expression. FH-deficient cells participate in the biosynthesis and degradation of haem and rely on purine recycling for nucleotide biosynthesis. Excess fumarate can be used to generate argininosuccinate using the reverse activity of ASL with arginine. Fumarate releases mitochondrial DNA (mtDNA) into the cytoplasm via mitochondrial-derived vesicles and then triggers the activation of the cGAS/STING/TBK1 pathway. The accumulation of tumor-derived fumarate in the TME can succinate ZAP70, resulting in the inhibition of TCR signaling and CD8^+^ T cells. Increased fumarate causes high levels of protein succination. Succination of KEAP1 enhances NRF2 stability and promotes tumor progression through multiple pathways. Succination of IRP2, PTEN, GSDMD, GSH, and other proteins affects tumor cells by altering their downstream pathways. Succination of key proteins in the respiratory chain leads to the dysfunction of complex I, while fumarate accumulation can also directly inhibit complex II.

In FH-deficient tumors, the succination of Kelch-like ECH-associated protein 1 (KEAP1) impaired the ubiquitin E3 ligase complex, which enhanced the stability of nuclear factor (erythroid-derived 2)-like 2 (Nrf2) [156]. NRF2 drives tumor progression, metastasis, and resistance to therapy by acting as a master regulator of cellular antioxidant responses. NRF2 and its target genes play a role in promoting tumor through continuous proliferation signal transduction, inhibiting apoptosis, senescence prevention, promoting angiogenesis, and EMT. However, whether NRF2 activation in tumors is beneficial or detrimental depends on environment and types of tumors [157]. In FH-inactivated tumor cells, fumarate can also facilitate ferritin mRNA translation through the succinylation of iron regulatory protein 2 (IRP2), and an increase in ferritin can promote forkhead box protein M1 (FOXM1) expression and cell proliferation [158]. The succination of PTEN occurs in FH-deficient cells. The succination of PTEN abolished its suppression on PI3K/AKT pathway, thus promoting tumorigenesis [159]. Moreover, the succination of GSDMD blocks pyroptosis, thereby promoting tumor cell survival [160]. In addition, studies have shown that fumarate induces the succination of primary units of proteins in the iron–sulfur cluster biogenesis family, resulting in respiratory chain complex I dysfunction, and fumarate accumulation can directly inhibit complex II. Therefore, FH-deficient cells exhibit mitochondrial respiratory disorders [161]. Fumarate interferes with iron chelation to dose-dependently inhibit of ACO2 activity through succination, which affects the TCA cycle (Fig. 5) [162].

The fumarate-mediated succination process is highly specific and occurs exclusively in tumor types with fumarate accumulation, distinguishing it from normal tissues and other tumor types. This unique mechanism is considered a candidate pathway for oncogenesis. By succinating multiple targets such as KEAP1, IRP2, PTEN, and GSDMD, fumarate confers a growth advantage to tumor cells and exerts nonmetabolic, tumorigenic effects.

### Metabolic rewiring and oxidative stress

FH-deficient renal cancer is highly glycolytic, exhibiting glucose-dependent growth, increased production rates of lactate, enhanced HIF-1α expression, and excess Glut1 and LDHA expression [163]. Changes in mitochondrial DNA are suggested to underlie the transition to aerobic glycolysis. The inactivation of FH drives a metabolic conversion to aerobic glycolysis, causing reduced levels of AMPK and tumor suppressor p53, consequently, defects in iron metabolism, and cytoplasmic iron deficiency can increase HIF-1α expression. A decrease in AMPK activity can also activate fatty acid and protein biosynthesis pathways and promote oncogenesis in FH-deficient cells [164]. Complete glucose oxidation in the mitochondria is limited, and glutamine becomes the main carbon source in FH-deficient cells. Furthermore, FH-deficient cells participate in the biosynthesis and degradation of haem to capitalize on the accumulated TCA cycle metabolites and generate NADH required to meet their own survival needs [165]. In addition, excess fumarate can use the inverse activity of argininosuccinate lyase (ASL) with arginine to generate argininosuccinate, which is a metabolite of the urea cycle and is thought to be produced from citrulline and aspartate. Because of this altered metabolic pathway, FH-deficient cells become dependent on arginine, and blocking the pathway specifically reduces their proliferation [166]. Moreover, fumarate accumulation impedes the biosynthesis of purines. FH-deficient cells rely on purine salvage for nucleotide biosynthesis to promote proliferation, rather than de novo purine biosynthesis. Inhibiting the purine salvage pathway through genetic alterations or drugs reduces the growth of HLRCC [167].

Fumarate also disrupts redox homeostasis. Although fumarate stabilizes the antioxidant factor NRF2, the FH-inactivating mutation causes increases glucose-mediated cellular ROS, which is critical for the stabilization of HIF-1α [168]. The mechanism may involve the generation of succinated GSH (GSF) through succination of the antioxidant GSH. GSH reductase can deplete NADPH and promote an increase in ROS using GSF as an alternative substrate (Fig. 5) [169].

### Fumarate and immunity

Increased intracellular fumarate induces early changes in mitochondrial morphology and facilitates mitochondrial DNA released into the cytoplasm via mitochondrial-derived vesicles. These alterations activate the cGAS/STING/TBK1 pathway and subsequent innate immune response [170]. In addition, Tumor-cell-derived fumarate accumulation in the TME can succinate zeta chain of T cell receptor-associated protein kinase 70 (ZAP70), which is a tyrosine kinase crucial in initiating CD8^+^ T cell activation. ZAP70 function is abrogated, and TCR signaling is disrupted, leading to the suppression of CD8^+^ T cell activation and antitumor efficacy (Fig. 5) [171].

## Malate and OAA: The Maintainers of Redox Homeostasis

In TCA cycle, malate is dehydrogenated to form OAA and start the next cycle. In the cytoplasm, aspartate is catalyzed by GOT1 to produce OAA, followed by MDH1 catalyzing the reduction of OAA to malate. Malate is transported into mitochondria in exchange for α-KG. Inside mitochondria, malate is oxidized to OAA by MDH2, and, subsequently, OAA is converted to aspartate by transamination catalyzed by GOT2. Glutamate donates the amino group and produces α-KG. Aspartate then enters the cytoplasm. The process is defined as the malate–aspartate shuttle, which plays a crucial role in coordinating glucose and amino acid metabolism and supporting proliferation of various types of tumor cells [172,173].

In human pancreatic ductal adenocarcinoma, GOT1 converts glutamine-derived aspartate to OAA, which generates malate and subsequent pyruvate, increasing the NADPH/NADP^+^ ratio and maintaining redox homeostasis in tumor cells. This process is mediated by oncogenic Kirsten rat sarcoma viral oncogene homolog (KRAS), promoting the rapid growth of tumors [174]. GOT1 has been found to be up-regulated in lung and breast cancers but down-regulated in brain and colorectal cancers, suggesting its complex function in cancer metabolism [175].

Malate can also be catalyzed by malic enzyme (ME) to generate pyruvate, accompanied by the generation of intracellular reducing equivalent NADPH. Knockdown of ME in gastric cancer cells induced a significant increase in ROS and apoptosis in the case of oxidative stress [176].

In addition, OAA supplementation showed potent antitumor effects in hepatocellular carcinoma cell lines by inducing apoptosis and ROS accumulation. OAA enhanced OXPHOS and decreased the expression of glycolysis-related enzymes HK, PFK, and LDH by inhibiting the Akt/HIF signaling pathway. The inhibition of glycolysis significantly impaired the survival of tumor cells [177].

The role of the 2 remaining intermediates, aconitate and isocitrate, in tumors is unclear and remains to be elucidated with further studies.

## Therapeutic Implications

The metabolites of the TCA cycle and their associated metabolic enzymes play critical roles in tumor biology, providing valuable insights for researchers exploring therapeutic strategies related to cancers. Here, we focus on pharmacological agents that specifically target the TCA cycle and elucidates the underlying mechanisms.

### Targeting IDH mutations

Enasidenib (AG-221/CC-90007) was a selective inhibitor of mutant IDH2 (mIDH2), as the first-in-class small-molecule inhibitor to enter clinical trials. The outcome of the first phase 1/2 study in patients with relapsed or refractory (R/R) AML was positive. The overall response rate was 40.3%, and the median response duration is 5.8 months, with a median overall survival of 9.3 months and 19.3% among all patients attaining complete remission (NCT01915498). Enasidenib improved clinical response by inducing tumor cells differentiation. However, in this trial, IDH-inhibitor-associated differentiation syndrome occurred in approximately 12% of patients treated with enasidenib, which was potentially lethal and required prompt recognition and management [178]. Whereafter, the US Food and Drug Administration (FDA) approved it to apply to mIDH2 R/R AML. Enasidenib was also well tolerated and induced durable remissions in older population of patients with newly diagnosed AML (NCT01915498). Current clinical studies combined enasidenib with intensive chemotherapy (NCT02632708) or azacitidine (NCT02677922 and NCT03683433), improving outcomes for patients with AML. The application of drugs also extended to mIDH2 myelodysplastic syndromes (NCT01915498).

Ivosidenib (AG-120) serves as a small-molecule inhibitor of mIDH1. Ivosidenib markedly decreased the concentration of D-2-HG in IDH1-mutant low-grade glioma [179]. In addition, ivosidenib induced the durable remissions in IDH1-mutated (R/R) AML (NCT02074839) and newly diagnosed IDH1-mutant AML (NCT02074839). Ivosidenib was well tolerated in patients with IDH1-mutant advanced cholangiocarcinoma (NCT02073994) and significantly improving progression-free survival in patients with IDH1-mutant, chemotherapy-refractory cholangiocarcinoma (NCT02989857). FDA approved ivosidenib used for patients with advanced unresectable or metastatic hepatocellular IDH1-mutated cholangiocarcinoma. The combination of ivosidenib and intensive chemotherapy [180] or azacitidine [181] (NCT02677922) also achieved better outcomes. Ivosidenib exerted low toxicity and remarkable efficacy in patients with advanced chondrosarcoma (NCT02073994).

Vorasidenib (AG-881) is proved to be an effectively oral brain-penetrant inhibitor targeting mIDH1 and mIDH2 for treatment of low-grade gliomas. In a perioperative phase 1 study, investigators measured the concentration of 2-HG in surgically resected tumor tissues from patients with IDH-mutated low-grade gliomas. The analysis revealed that the mean 2-HG concentration in tumors from patients without receiving vorasidenib before surgery was a 154.9 μg·g^−1^, while the mean concentrations in patients who received vorasidenib at doses of 10 or 50 mg q.d. (quaque die) before surgery were 67.5 and 8.9 μg·g^−1^, respectively (NCT03343197). After comparing data from untreated patients with patients receiving vorasidenib of 50 mg q.d., it was found that the latter had a more than 90% decrease in 2-HG levels (calculated value, 92.6%) [182]. A first-in-human phase 1 trial in patients with recurrent or progressive gliomas following standard therapy found that vorasidenib demonstrated favorable safety in the glioma cohort and showed preliminary antitumor effects (NCT02481154). In another double-blind phase 3 trial, vorasidenib significantly improved progression-free survival in patients with grade 2 IDH-mutant gliomas and significantly improved the time to next intervention (NCT04164901).

Olutasidenib (FT-2102) showed meaningful clinical activity (NCT02719574) and was recently approved by FDA for the treatment of patients with IDH1 mutation R/R AML. Patients with recurrent or progressive IDH1-mutant glioma well responded to DS-1001 with a favorable brain distribution of it (NCT03030066). Moreover, some inhibitors had shown other problems during clinical trials, which did not support further trials. In the phase 1 study, the mIDH1 inhibitor BAY1436032 demonstrated the clinical benefit of safety and modest effectiveness in IDH1-mutant AML. Nevertheless, the overall response rate was low, and it did not show complete inhibition at highest dose (NCT03127735). Besides, on account of potentially narrow therapeutic window of IDH305, the phase 1 study in patients with IDH1-mutant AML or myelodysplastic syndrome was prematurely halted (NCT02381886). It was reported that many inhibitors targeting IDH1 (quinolinone derivative [183], GSK321 [184], etc.) or IDH2 (CP-17 [185], AGI-6780 [186], etc.), which have been developed to apply to tumors, but not in clinical trials yet.

### Targeting ACLY

A growing number of evidence emphasizes the crucial role of ACLY, conferring tremendous therapeutic potentials targeting cancer to this enzyme. ACLY inhibitors, which are previously developed for reducing levels of low-density lipoprotein cholesterol, are recognized as promising anticancer strategies recently. ACLY inhibitors include naturally derived inhibitors and synthetic compound.

The first discovered natural inhibitor was (−)-hydroxycitric acid (HCA), which was used to research its effects on fatty acid oxidation when supplemented to endurance-trained humans in the clinical trial. Various studies have validated the antitumor effects of HCA. In chronic myelogenous leukemia, HCA was capable of inhibiting tumors growth in vitro and in xenograft models through activating AMPK and mTOR pathway [187]. The combination of HCA and α-lipoic acid leads to remarkable retardation of tumor growth in mouse syngenic cancer models of bladder carcinoma, melanoma, and lung carcinoma [188]. The results in a case of an 80-year-old female with pancreas ductal adenocarcinoma who treated with gemcitabine plus α-lipoic acid and HCA were also highly promising [189]. Another natural inhibitor that has been studied in tumors is cucurbitacin B. Cucurbitacin B can exert anticancer effects by modulating various signaling pathways, such as the Janus kinase (JAK)/STAT [190], TLR4/NLRP3/GSDMD [191], PI3K/Akt/mTOR [192], heat shock protein family A (Hsp70) member 5 (GRP78)/FOXM1/kinesin family member 20A (KIF20A) [193], and NF-κB [194]. Meanwhile, a study in prostate cancer indicated that cucurbitacin B inhibited the phosphorylation of ACLY in a dose-dependent way, which was then confirmed as a direct target of cucurbitacin B and abrogated its antitumor effects [195].

Bempedoic acid (ETC-1002) was the only one synthetic inhibitor approved as the monotherapy for patients with hypercholesterolemia, based on the positive outcomes in clinical trials (NCT02993406 and NCT02666664). Bempedoic acid dramatically suppressed the metastasis of the colorectal cancer with the IGF1–HOXA13–IGF1R-positive feedback loop, when combining with an IGF1R inhibitor [196]. In addition, the combination of bempedoic acid and cyclin-dependent kinase 4/6 (CDK4/6) inhibition limited tumor cells growth and invasion [197]. Another inhibitor NDI-091143 induced the apoptosis in thyroid cancer and sensitized the therapeutic effect of sorafenib [198].

Although many inhibitors have been developed, only a minority have been applied to tumors. Therefore, further studies in vitro and human clinical trials are also required to evaluate the existing ACLY inhibitors as antitumor therapies.

### Targeting GLS

Highly selective inhibitors of the GLS, a crucial enzyme in tumor anabolism, have been developed over the past 2 decades. Among them, telaglenastat (CB-839) is the only one advancing into and completing clinical trials. CB-839 displayed the antitumor activity in various types of tumors, for instance, the triple-negative breast cancer [199], ovarian carcinoma [200], hepatocellular carcinoma [201], and lung adenocarcinoma [202]. A phase 1 clinical trial in patients with the phosphatidylinositol-4,5-bisphosphate 3-kinase catalytic subunit alpha-mutant colorectal cancer indicated that the combination of CB-839 and 5-FU suggested a trend of clinical benefit and might be a promising treatment (NCT02861300). In another open-label phase 1 trial, CB-839 plus either everolimus or cabozantinib showed marked antitumor activity and tolerability in patients with metastatic renal cell carcinoma (NCT02071862). In the phase 2 ENTRATA trial, CB-839 plus everolimus showed improvement in progression-free survival of patients with metastatic renal cell carcinoma previously treated with tyrosine kinase inhibitors and checkpoint inhibitors (NCT03163667). In addition, CB-839 demonstrated potential for application in patients with hematologic tumors (NCT02071927 and NCT02071888).

Other inhibitors that have been validated to be effective in tumors are IPN60090 (IACS-6274), 968, and BPTES. IPN60090 induced inhibition of tumors in non-small-cell lung cancer patient-derived xenograft mouse model and showed obviously better efficacy in combination with the dual mTORC1/2 inhibitor [203]. An open-label phase 1 trial investigating the safety, pharmacokinetics, and antitumor effect of IPN60090 in patients with advanced solid tumors was ongoing (NCT05039801). Moreover, 968 was proved to boost the immune response when combining with anti-PD-L1 against ovarian cancer [204]. BPTES specifically targeted GLS and blunted tumor progression in MYC-dependent hepatocellular carcinoma and lymphoma [205].

Another effective way is to antagonize glutamine. Sirpiglenastat (DRP-104) was found as a prodrug of 6-diazo-5-oxo-l-norleucine (DON), a glutamine antagonist, and it was preferentially bioactivated to DON in tumors [206]. Tumors treated with DRP-104 disrupted tumor anabolism and typical metabolic pathways, such as reduced glutamine flux into the TCA cycle. It also exerted antitumor immune effects by increasing and activating immune cells [207]. The use of DRP-104 resulted in significant reductions of tumor growth in several in vivo models [208]. A phase 1b/2 study of DRP-104 combining with durvalumab (an immune checkpoint inhibitor) in patients with advanced stage fibrolamellar hepatocellular carcinoma was ongoing (NCT06027086).

### Targeting α-KG

CPI-613 (devimistat) is widely used in the treatment of tumors as an inhibitor of PDH and α-KGDH. CPI-613 exerted antitumor activity through ROS-associated apoptosis, as well as enhanced autophagy and inhibition of lipid metabolism via the AMPK-Acetyl-coenzyme A carboxylase (ACC) signaling in pancreatic cancer [209]. Furthermore, CPI-613 dramatically attenuated the progression of melanoma and simultaneously improved the therapeutic efficacy of anti-PD-1 immunotherapy through increasing PD-L1 expression mediated by AMPK–adenosine 3′,5′-monophosphate response element-binding protein–activating transcription factor 3 (ATF3) signaling [210].

Clinical trials of CPI-613 applying to different tumors types were conducted successively. The result of a phase 1 study of CPI-613 in patients with advanced hematologic malignancies demonstrated its limitation of mitochondrial functions and clinical benefit to heavily pretreated patients (NCT01034475). In phase 1/2/3 trial of CPI-613 combining with cytarabine and mitoxantrone in patients with AML, the combination treatment strategy exhibited lower toxicity and promising clinical outcomes in older patients and those with poor-risk cytogenetics (NCT02484391 and NCT03504410). CPI-613 was combined with other drugs to treat metastatic pancreatic cancer (NCT01835041 and NCT03504423) and biliary tract cancer [211].

Targeting citrate transporters (SLC13A5 and SLC25A1) are also considered an effective approach to restraining tumors. These inhibitors exerted selective cytotoxicity to tumors, such as hepatocellular carcinoma [212] and breast cancer [213], and did not affect normal cells. Further development is needed to validate the antitumor efficacy and toxicity of inhibitors of the citrate transporters in humans. The mutations of FH and SDH are challenging to be inhibited, but there are several agents manifesting the suppression activity for these enzymes [214]. In addition, supplement of citrate and α-KG has also shown potentials of opening new avenues for clinical cancer therapy [215].

## Conclusions and Future Perspectives

The TCA cycle is a key metabolic pathway for the utilization of glucose, amino acids, and fatty acids by organisms and is the bioenergetic central of metabolism, biosynthesis, and redox homeostasis. Although tumor cell metabolic reprogramming has led to the predominance of aerobic glycolysis, tumor cells still require the TCA cycle for a continuous supply of precursors for anabolic reactions such as lipids, proteins, and nucleic acids. Intermediate metabolites of the TCA cycle also vary across different types of tumors due to genetic mutations or altered cellular metabolism.

The roles of several metabolites, including citrate, α-KG, succinyl-CoA, succinate, and fumarate, in tumorigenesis and tumor progression have been progressively elucidated through extensive research (Table). However, there are still unanswered questions that require further investigation. For instance, new mechanisms by which these metabolites affect tumors need to be discovered, and the roles of other metabolites such as malate, isocitrate, and OAA in tumor biology remain to be fully explored. Continued studies are necessary to shed light on these aspects and enhance our understanding of metabolic dysregulation in cancer.

Indeed, the relationship between TCA cycle metabolites and tumors displays considerably complicated, in the heterogeneity of tumors and the intricate regulation of metabolites within the TME, which implies that the metabolic processes in cancer cells are highly complex and can differ between various tumor types and even within individual tumors. Further investigations are necessary to unravel the specific mechanisms and regulatory pathways that govern the interplay between TCA cycle metabolites and tumor biology. Such insights will be critical for developing more effective therapeutic strategies targeting tumor metabolism.

Currently, several small-molecule drugs targeting crucial molecules in the TCA cycle have been investigated in tumor-related clinical trials, and some have shown promising results. The exploration of new therapeutic targets within the TCA cycle and the development of associated drugs are areas that warrant further research. Moreover, selecting safer and more effective drugs with minimal side effects from the pool of existing compounds that have demonstrated efficacy against tumors in vitro is a promising direction for clinical trials. This approach requires careful evaluation and optimization to ensure the successful translation of laboratory findings into clinically viable treatments for patients with cancer. However, cancer cells tend to adapt and develop drug resistance through compensatory pathways. Therefore, a thorough understanding of the alterations in the TCA cycle in cancer will provide new insights into the development of new and effective therapeutic approaches.
